# Functional brain activity in patients with amnestic mild cognitive impairment: an rs-fMRI study

**DOI:** 10.3389/fneur.2023.1244696

**Published:** 2023-08-22

**Authors:** Jinhuan Yue, Sheng-wang Han, Xiao Liu, Song Wang, Wei-wei Zhao, Li-na Cai, Dan-na Cao, Jeffrey Zhongxue Mah, Yu Hou, Xuan Cui, Yang Wang, Li Chen, Ang Li, Xiao-ling Li, Guanhu Yang, Qinhong Zhang

**Affiliations:** ^1^Shenzhen Frontiers in Chinese Medicine Research Co., Ltd., Shenzhen, China; ^2^Department of Acupuncture and Moxibustion, Vitality University, Hayward, CA, United States; ^3^Department of Third Rehabilitation Medicine, Second Affiliated Hospital of Heilongjiang University of Chinese Medicine, Harbin, China; ^4^Department of Pediatrics, First Affiliated Hospital of Heilongjiang University of Chinese Medicine, Harbin, China; ^5^Department of Radiology, Longhua Hospital, Shanghai University of Traditional Chinese Medicine, Shanghai, China; ^6^MSD R&D (China) Co., Ltd., Beijing, China; ^7^Graduate School of Heilongjiang University of Chinese Medicine, Harbin, China; ^8^Division of CT and MRI, First Affiliated Hospital of Heilongjiang University of Chinese Medicine, Harbin, China; ^9^Department of Gynecology, Harbin Traditional Chinese Medicine Hospital, Harbin, China; ^10^Confucius Institute for TCM, London South Bank University, London, United Kingdom; ^11^Sanofi-Aventis China Investment Co., Ltd., Beijing, China; ^12^Department of Specialty Medicine, Ohio University, Athens, OH, United States; ^13^Department of Acupuncture and Moxibustion, Heilongjiang University of Chinese Medicine, Harbin, China

**Keywords:** amnestic mild cognitive impairment, resting-state functional magnetic resonance imaging, regional homogeneity, amplitude of low-frequency fluctuation, imaging marker

## Abstract

**Background:**

Amnestic mild cognitive impairment (aMCI) is an early stage of Alzheimer’s disease (AD). Regional homogeneity (ReHo) and amplitude of low-frequency fluctuation (ALFF) are employed to explore spontaneous brain function in patients with aMCI. This study applied ALFF and ReHo indicators to analyze the neural mechanism of aMCI by resting-state functional magnetic resonance imaging (rs-fMRI).

**Methods:**

Twenty-six patients with aMCI were included and assigned to the aMCI group. The other 26 healthy subjects were included as a healthy control (HC) group. Rs-fMRI was performed for all participants in both groups. Between-group comparisons of demographic data and neuropsychological scores were analyzed using SPSS 25.0. Functional imaging data were analyzed using DPARSF and SPM12 software based on MATLAB 2017a. Gender, age, and years of education were used as covariates to obtain ALFF and ReHo indices.

**Results:**

Compared with HC group, ALFF decreased in the left fusiform gyrus, left superior temporal gyrus, and increased in the left cerebellum 8, left inferior temporal gyrus, left superior frontal gyrus (BA11), and right inferior temporal gyrus (BA20) in the aMCI group (*p* < 0.05, FWE correction). In addition, ReHo decreased in the right middle temporal gyrus and right anterior cuneiform lobe, while it increased in the left middle temporal gyrus, left inferior temporal gyrus, cerebellar vermis, right parahippocampal gyrus, left caudate nucleus, right thalamus, and left superior frontal gyrus (BA6) (*p* < 0.05, FWE correction). In the aMCI group, the ALFF of the left superior frontal gyrus was negatively correlated with Montreal Cognitive Assessment (MoCA) score (*r* = −0.437, *p* = 0.026), and the ALFF of the left superior temporal gyrus was positively correlated with the MoCA score (*r* = 0.550, *p* = 0.004). The ReHo of the right hippocampus was negatively correlated with the Mini-Mental State Examination (MMSE) score (*r* = −0.434, *p* = 0.027), and the ReHo of the right middle temporal gyrus was positively correlated with MMSE score (*r* = 0.392, *p* = 0.048).

**Conclusion:**

Functional changes in multiple brain regions rather than in a single brain region have been observed in patients with aMCI. The abnormal activity of multiple specific brain regions may be a manifestation of impaired central function in patients with aMCI.

## 1. Introduction

Alzheimer’s disease (AD), the most common type of dementia, is characterized by permanent and irreversible neurodegeneration. A previous study estimated that approximately 65.7 million people may experience dementia by 2030, and 115.4 million by 2050 globally (1). Therefore, it has become a serious public health concern worldwide (1). However, its etiology and pathogenesis remain unclear and optimal clinical treatment is lacking. More importantly, about two-thirds of patients diagnosed with moderate to severe disease missed the optimal intervention time. Amnestic mild cognitive impairment (aMCI) is recognized as an early stage of AD (2, 3). Therefore, early diagnosis, early intervention, and delay in disease progression are the main research directions for managing AD, which is of great significance for improving the quality of life of patients and reducing the burden on the family and society.

Regional homogeneity (ReHo) is a reliable resting-state functional magnetic resonance imaging (rs-fMRI) analysis modality for exploring local functional connectivity, which measures the similarity of the time series at rest between a given voxel and its adjacent voxel. It has been widely used in pathophysiological studies of aMCI and AD (4). A previous study reported that the accuracy of ReHo in differentiating patients with aMCI from healthy controls was 90.5%, with a sensitivity of 86.21% and specificity of 93.94% (5). Furthermore, ReHo alterations have been observed in patients with aMCI and are associated with cognitive and memory impairments (6, 7). A meta-analysis showed that aMCI is associated with abnormal ReHo in the default mode network (DMN), executive control network, visual network, and sensorimotor network, suggesting that functional defects and compensation coexist in these networks (8). Therefore, ReHo can be used as a sensitive imaging marker in functional imaging studies in patients with aMCI.

The amplitude of low-frequency fluctuations (ALFF) is a reliable data-driven measurement of the intensity of spontaneous neuronal activity in a local area of the brain. Both animal models and human studies have shown that ALFF reflects synchronous neural activity during rs-fMRI studies, has many similarities to fluctuations in neurophysiology, hemodynamics, and metabolic parameters, and can be used to characterize regional functional changes in patients (9, 10). Another previous study found that compared with healthy control subjects, ALFF in the left superior temporal gyrus, right middle temporal gyrus, right inferior parietal lobe, and right posterior central gyrus was decreased in patients with aMCI but increased in the left superior frontal gyrus and middle frontal gyrus (11). Studies have shown that increased frontal activation in patients with aMCI may be an effective compensatory supplement to brain resources, which may provide a better understanding of cognitive changes in aMCI. Therefore, rs-fMRI based on ALFF and ReHo analyses may provide a reference for the study of brain function in patients with aMCI. In this study, we applied both ALFF and ReHo indicators to comprehensively explore the central mechanism of aMCI by rs-fMRI and investigate additional options for early diagnosis and treatment schedule.

## 2. Methods and materials

### 2.1. Ethical approval

This study has been approved by the Medical Ethical Committee of The First Affiliated Hospital of Heilongjiang University of Chinese Medicine (HZYLLBA202012).

### 2.2. Participants

This prospective, cross-sectional study recruited aMCI patients from January 2021 to December 2022 from the Department of Neurology, the First Affiliated Hospital of Heilongjiang University of Chinese Medicine in accordance with the inclusion and exclusion criteria. According to the diagnostic criteria of aMCI proposed by Petersen (12) and Chinese experts on Prevention and Treatment of Cognitive Impairment (13), the subjects were divided into aMCI group and healthy control (HC) group.

### 2.3. Eligibility criteria

The inclusion criteria for aMCI group were as follows: (1) main complaint of aMCI was dysmnesia, which was confirmed by an informed person; (1) score of 24 ≤ Mini-Mental State Examination (MMSE) ≤ 27, 18 < Montreal Cognitive Assessment (MoCA) < 26, and Clinical Dementia Rating (CDR) = 0.5; (3) ability in daily life was not affected; (4) age ranging from 50 to 70 years; (5) right-handedness; (6) educational years >8 years or education level above junior high school; and (7) informed consent of the participants was provided.

The inclusion criteria for the HC group were as follows: (1) age, sex, and education level similar to those of the aMCI group; (2) right-handedness; (3) good ability in daily life without complaints of cognitive decline; (4) MMSE score > 27, MOCA score ≥ 26, and CDR score = 0; and (5) informed consent obtained from all participants.

The exclusion criteria were as follows: (1) Hachinski ischemic score > 4 and history of acute cerebrovascular disease in the past 3 months; (2) history of congenital, organic or functional diseases of the nervous system; (3) addictive disorders, such as drug or alcohol abuse; and (4) contraindications or claustrophobia on magnetic resonance imaging (MRI) scans.

### 2.4. MRI data acquisition

All subjects underwent conventional MRI, 3D-T1WI, and BOLD-fMRI. The scanning technician fixed the head of each participant using a sponge pad. The participants were asked to remain awake, breathe steadily, relax, and try not to think during the entire test process.

3D-T1WI scan: A three-dimensional fast scrambled phase gradient echo inversion recovery sequence (T1WI-3D-TFE-REF) was used to obtain sagittal T1WI, which included the whole brain and can provide a template for the segmentation of gray matter, white matter, and cerebrospinal fluid. The scanning parameters were as follows: repetition time (TR) = 6.7 ms, echo time (TE) = 3.0 ms, matrix = 256 × 256, field of vision (FOV) = 256 × 256 × 288 mm, scanning slices = 188 layers, slice thickness = l mm, slice gap = 0 mm, flip angle =8°, and scanning time = 4 min and 39 s (Table 1).

**Table 1:** Structural 3D and functional imaging sequence parameters.

Parameters	3D T_1_WI	BOLD-fMRI
Sequence	T_1_WI-3D-TFE-ref	FFE single-shot EPI
TR	6.7 ms	2000 ms
TE	3.0 ms	30 ms
FOV	256 × 256× 288 mm	220 × 220 × 143 mm
Matrix	256 × 256	64 × 64
Slices	188	36
Slices thickness	1 mm	3 mm
Slice gap	0 mm	1 mm
Flip angle	8°	90°
Slice time	4 min 39 s	6 min 6 s

TR, repetition time; TE, echo time; FOV, field of vision.

Rs-fMRI scan: Using the FFE single-shot EPI sequence, the scanning speed is fast, and all images can be acquired in tens of milliseconds through multiple echo chains with only one shot of an RF pulse, which can achieve the goal of instantaneous imaging. The scanning parameters: TR = 2,000 ms, TE = 30 ms, matrix = 64 × 64, FOV = 220 × 220 × 143 mm, scanning slices = 36 layers, slice thickness = 3 mm, slice gap = 1 mm, flip angle =90°, and scanning time = 6 min and 6 s (Table 1).

### 2.5. Flow of image data processing

Based on MATLAB 2017A platform DPARSF software data pre-processing, the steps are as follows: (1) Removing the data of the first 10 time points (volume), the signal may fluctuate at the beginning of the scan and the subject adapts to the process of the scanning environment, which will make the magnetic field unstable; therefore, it is necessary to remove the data of the first 10 time points to reduce or eliminate the effect of these data on the results; (2) Slice timing; (3) Head motion correction (realign), which removes subject data with translation >2 mm or rotation >2° in either direction; (4) Registration (coregister), which registers functional images into 3D structural images; (5) Segment: the 3D structure image is divided into gray matter, white matter, and cerebrospinal fluid; (6) Normalize: align the local spatial data of different subjects to the MNI standard space for statistical analysis. The voxel size after normalization was 3 mm × 3 mm × 3 mm; (7) Regression was used to remove six head movement parameters, white matter, and cerebrospinal fluid from the standardized data; (8) Smoothing was used to process the standardized functional images and reduce the difference produced by registration, while improving statistical power, using a Gauss core for FWHM = 6 mm × 6 mm × 6 mm; (9) Detrend, which is caused by many factors, such as the continuous running of the machine or the long static time of the subject, fatigue, etc.; (10) Filter, the low-frequency part of the Bold Signal Band, which mainly reflects the spontaneous activity of the brain, was selected to eliminate physiological signals (such as breathing and heartbeat) and random noise.

ReHo index calculation: Using DPARSF software, we calculated the Kendal concordance coefficient (KCC) between a voxel and the adjacent 26 voxels, and finally obtained the KCC value of each voxel, which made up the KCC brain map and ReHo diagram. KCC is used to measure the similarity of different voxel time series within an active functional area.

ALFF index calculation: Using DPARSF software with following steps: (1)all-time series without linear drift voxels were first band-pass filter at 0.01–0.08 Hz; (2) above results were fast Fourier transform, and the required power spectrum was obtained; (3) the power spectrum was squared; (4) mean of power spectrum was calculated as ALFF; (5) ALFF was divided by the mean of all voxels in the brain to obtain a standardized MALFF.

### 2.6. Statistical analysis

#### 2.6.1. Demographic and neuropsychological scale analysis

SPSS 25.0 software was used to analyze the demographic and neuropsychological scale scores of the two groups of subjects. The measurement data were presented as mean ± standard deviation, chi-square test was used for gender comparison between the two groups, two-sample *t*-test was used for age and years of education between the two groups, and *p* < 0.05 was used as the test level, with statistical significance. Pearson’s partial correlation analysis was used to assess the correlation between clinical cognitive scores and functional changes in the brain regions of patients with aMCI under the control of sex, age, and years of education, with a statistical significance of *p* < 0.05.

#### 2.6.2. Statistical analysis of brain MRI data

The functional data of ALFF and ReHo were analyzed using the SPM12 statistical parameter graph software. The results of the aMCI and HC groups were statistically analyzed using a one-sample *t-*test and FWE correction (*p* < 0.05). The adjusted functional areas of the two groups were made into a merged template using xjView software, and the two groups of independent samples were tested using SPM12 software with the merged template as the mask, with an initial voxel level of *p* = 0.001(unadjusted) when age, sex, and years of education were used as covariates. For multiple comparisons, the adjusted FWE levels of the clusters (*p* < 0.05) were statistically significant.

## 3. Results

### 3.1. General information

**Figure 1:**
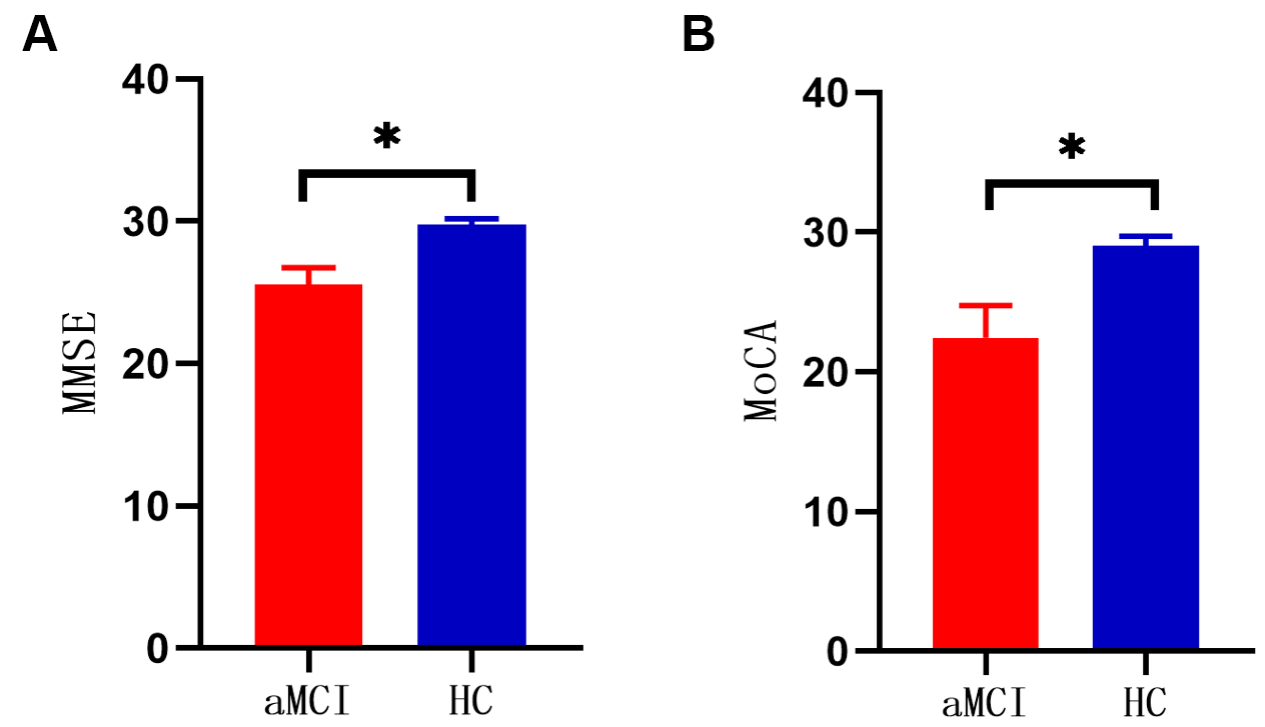
**(A)** Comparison of MMSE scores between two groups (*p < 0.001). **(B)** Comparison of MOCA scores between two groups (*p < 0.001).

There was no significant difference between the two groups in terms of demographic data such as age, sex, and years of education (*p* > 0.05). There were statistically significant differences in the neuropsychological scores of the MMSE and MoCA (*p* < 0.05) (Table 2; Figure 1).

**Table 2:** Comparison of demographic and neuropsychological scores between aMCI and HC groups.

Items	Age	Gender		Education	MMSE	MoCA
		Male	Female			
aMCI	62.00 ± 2.73	14	12	13.08 ± 2.00	25.58 ± 1.17	22.46 ± 2.30
HC	60.58 ± 3.88	14	12	13.54 ± 1.90	29.77 ± 0.43	29.04 ± 0.66
*p* value	*p* = 0.133	*P* > 0.05		*p* = 0.398	*p* < 0.001	*p* < 0.001

aMCI, amnestic mild cognitive impairment; HC, healthy control; MMSE, Mini-Mental State Examination; MoCA, Montreal Cognitive Assessment.

### 3.2. ALFF index

**Figure 2:**
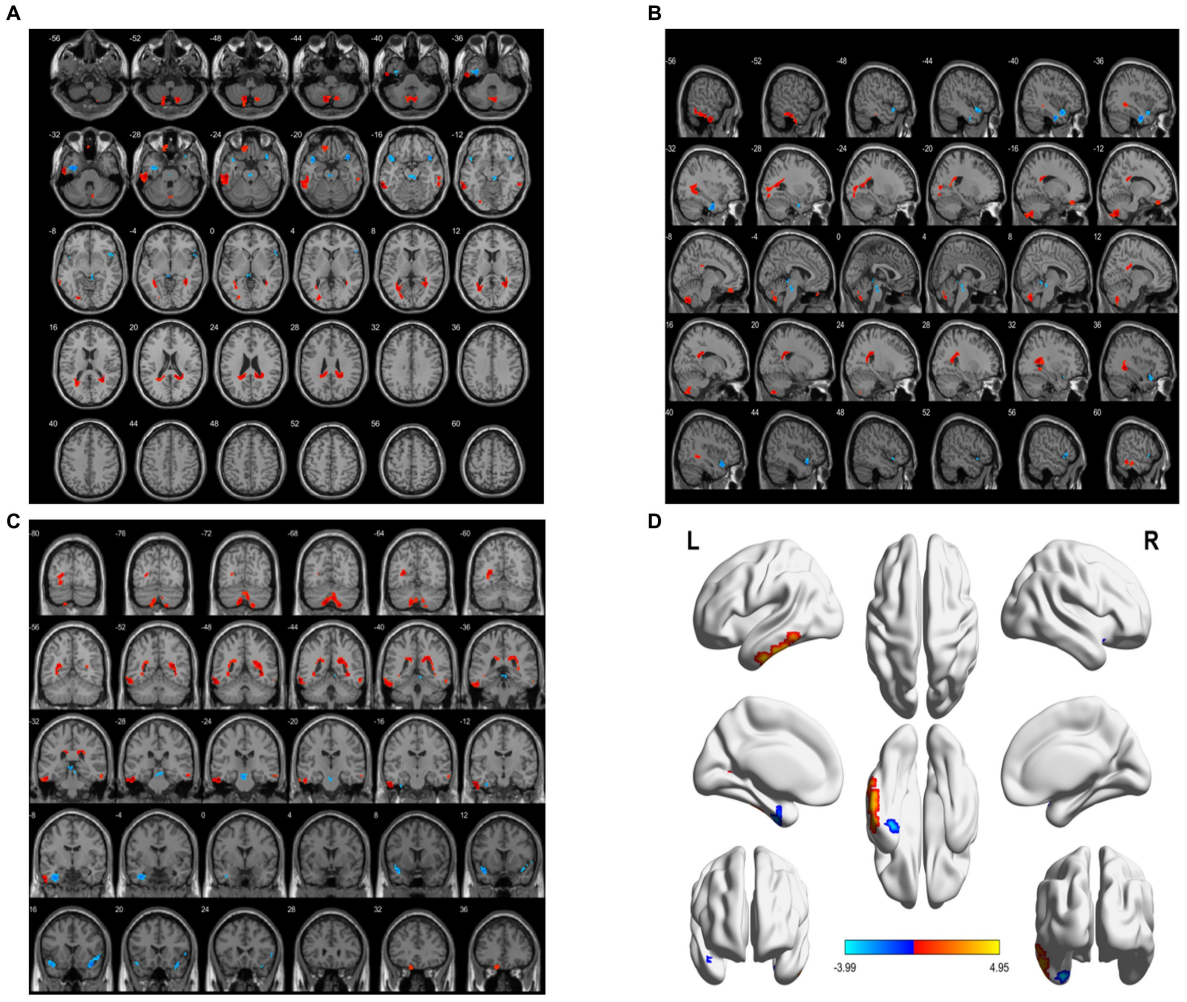
Brain area maps of ALFF changes in brain regions of the aMCI group compared to HC group. **(A)** Axial map, **(B)** Sagittal map, **(C)** Coronal map, **(D)** 3D map, ALFF = amplitude of low- frequency fluctuations.

Compared with the HC group, the decreased ALFF in the aMCI group was in areas of the left fusiform gyrus, left superior temporal gyrus, whereas the areas of increased ALFF were in the left cerebellum 8, left inferior temporal gyrus, left superior frontal gyrus (BA11), and right inferior temporal gyrus (BA20) (*p* < 0.05, FWE correction) (Table 3; Figure 2).

**Table 3:** Brain areas changed by ALFF in aMCI group compared with HC group.

Number of voxels	Brain areas	Brodmann area	*T* (peak intensity)	Peak MNI coordinates		
				*X*	*Y*	*Z*
217	Cerebelum_8_L	–	4.5214	−12	−66	−48
223	Temporal_Inf_L	–	4.9529	−66	−45	−15
75	Fusiform_L	–	−3.9898	−36	−6	−30
51	Frontal_Sup_Orb_L	BA11	3.2516	−15	39	−21
58	Temporal_Pole_Sup_L	–	−3.7755	−39	12	−21
50	Temporal_Inf_R	BA20	3.8013	60	−42	−15

ALFF, amplitude of low-frequency fluctuation; aMCI, amnestic mild cognitive impairment; HC, healthy control.

### 3.3. ReHo index

**Figure 3:**
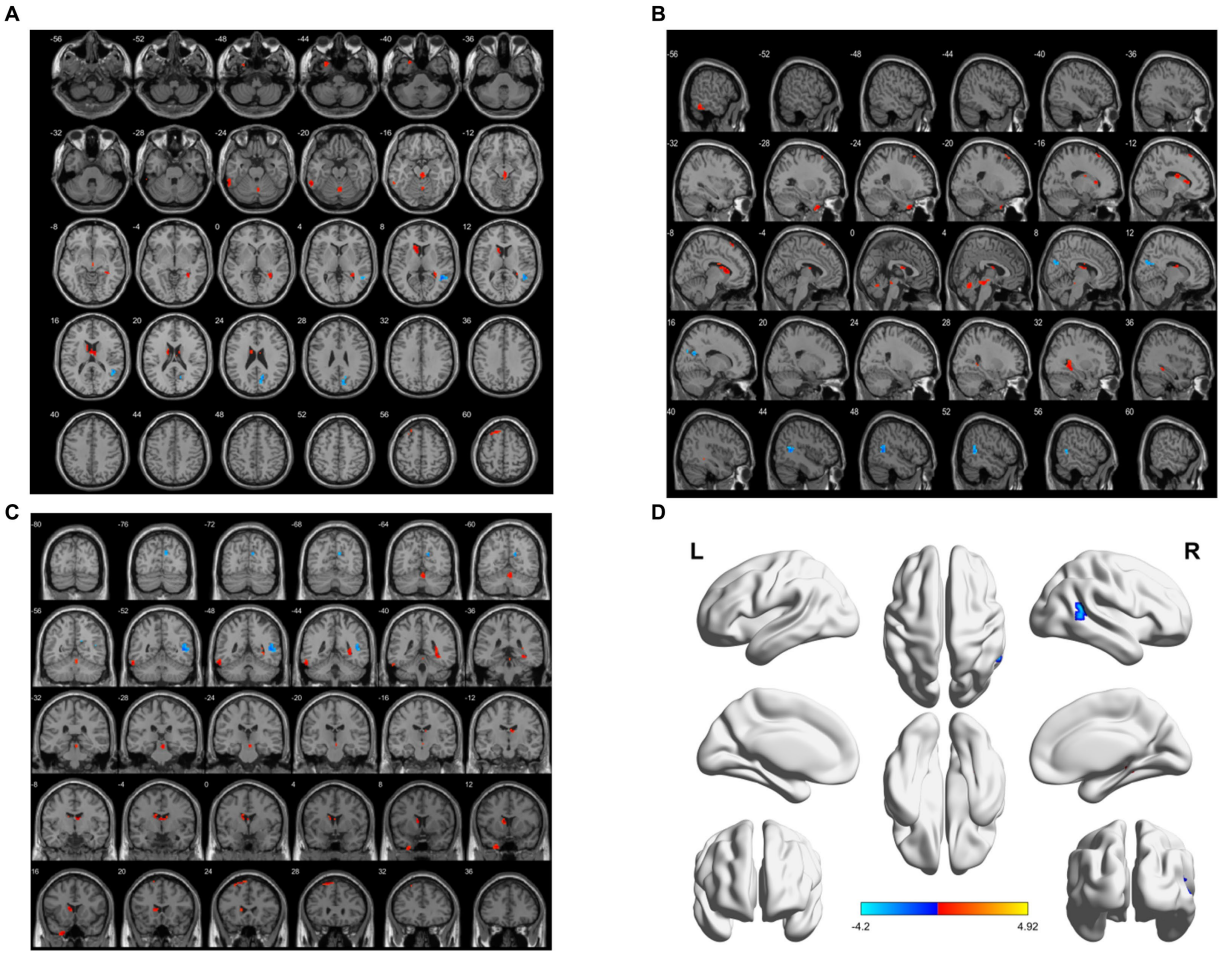
Brain area maps of ReHo changes in the brain regions of the aMCI group compared to HC group. **(A)** Axial map, **(B)** Sagittal map, **(C)** Coronal map, **(D)** 3D map, ReHo regional homogeneity.

ReHo decreased in the right middle temporal gyrus and right anterior cuneiform lobe. However, ReHo-enlarged areas were the left middle temporal gyrus, left inferior temporal gyrus, cerebellar vermis, right parahippocampal gyrus, left caudate nucleus, right thalamus, and left superior frontal gyrus (BA6) (*p* < 0.05, FWE correction) (Table 4; Figure 3).

**Table 4:** Brain areas changed by ReHo in aMCI group compared with HC group.

Number of voxels	Brain areas	Brodmann area	*T* (peak intensity)	Peak MNI coordinates		
				*X*	*Y*	*Z*
34	Temporal_Pole_Mid_L	–	4.1485	−21	15	−42
33	Temporal_Inf_L	–	3.9801	−60	−48	−21
31	Vermis_4_5	–	3.2976	3	−60	−18
52	ParaHippocampal_R	–	3.8062	33	−39	−6
60	Temporal_Mid_R	–	−3.9195	45	−51	9
61	Caudate_L	–	4.2185	−6	9	12
34	Thalamus_R	–	4.9203	9	−9	18
31	Precuneus_R	–	−3.6208	15	−63	24
32	Frontal_Sup_L	BA6	4.2669	−15	27	63

ReHo, regional homogeneity; aMCI, amnestic mild cognitive impairment; HC, healthy control.

### 3.4. Correlation between imaging parameters and clinical scale scores

**Figure 4:**
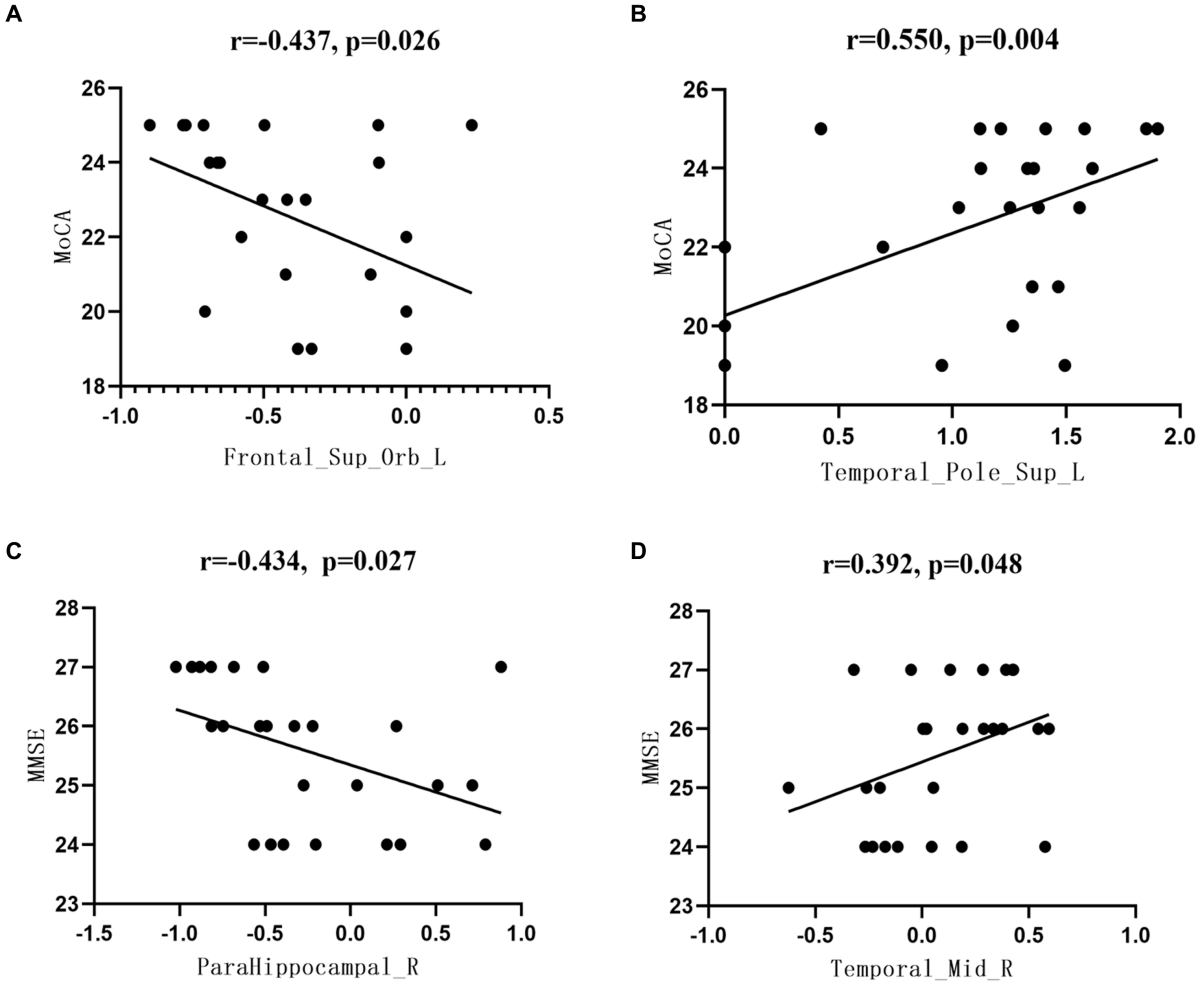
Correlation between the functional index data of brain regions and the neuropsychological scale scores. **(A)** Correlation analysis between Frontal_Sup_Orb_LALFF values and MoCA scores, **(B)** Correlation analysis between Temporal_Pole_Sup_L ALFF values and MoCA scores, **(C)** Correlation analysis between ParaHippocampal R ReHo values and MMSE scores, **(D)** Correlation analysis between Temporal_Mid_R ReHo values and MMSE scores.

In this study, there was a significant difference between the aMCI and HC groups in brain area, MMSE, and MoCA scores. There was a significant negative correlation between the left superior frontal gyrus ALFF and MoCA scores in the aMCI group (*r* = −0.437, *p* = 0.026), while in the left temporal pole, the ALFF of the superior temporal gyrus was positively correlated with the MoCA score (*r* = 0.550, *p* = 0.004); and the ReHo of the right parahippocampal gyrus were negatively correlated with MMSE score (*r* = −0.434, *p* = 0.027). There was a significant positive correlation between ReHo and MMSE scores (*r* = 0.392, *p* = 0.048) (Figure 4).

## 4. Discussion

Rs-fMRI explores brain function by detecting BOLD signals in a low-frequency band, which reflects the spontaneous activity of the brain. Among the most widely used rs-fMRI indices in clinical research, the methods used to analyze local spontaneous functional activity of the brain include ReHo (14, 15) and ALFF (16, 17). This study investigated the changes in ReHo and ALFF in the aMCI and HC groups and their physiological significance, which can provide objective evidence for aMCI identification.

### 4.1. Analysis of ALFF index change in patients with aMCI

ALFF is a repeatable, non-invasive, data-driven method for analyzing regional spontaneous neuronal activity at rest, reflecting the intensity of local regional spontaneous brain activity (18). A previous study used dynamic low-frequency amplitudes and dynamic low-frequency piece-wise amplitudes to evaluate the temporal variability of local brain activity in patients with mild cognitive impairment (MCI) (19). There were significant differences in ALFF between the posterior cerebellar lobe and the middle temporal lobe between the cognitive impairment group and HC group (19). This abnormal pattern may impair the ability of cerebellum and temporal lobe to participate in cerebellar circuits and DMN (19). Based on fMRI technology, one study employed ALFF to observe changes in resting brain function of working memory in patients with MCI (9). The ALFF values of the thalamus and other brain regions in patients with cognitive impairment were lower with those in the HC group (9). This study provides new insights into the neural mechanism underlying the working memory deficits in patients with MCI (9). An other study investigated changes in brain function in patients with early and late MCI (20). Significant differences in ALFF were found between early MCI, late MCI, and normal controls (NC) (20). Compared with NC, ALFF decreased linearly in the posterior cingulate cortex, anterior cuneiform lobe, right lingular gyrus, and thalamus (NC > early MCI > late MCI) but increased linearly in the right parahippocampal gyrus (NC < early MCI < late MCI) (20). In addition, ALFF changes in many regions were significantly associated with cognitive performance as measured by MMSE in patients with early and late MCI but not in healthy controls (20). Therefore, these findings suggest that an abnormal ALFF may serve as an imaging marker for the early diagnosis of MCI and provide new biological insights into functional neurological diseases (20).

A decrease in the ALFF in the brain region indicates a decrease in the spontaneous activity of neurons. In this study, compared with the HC group, the aMCI group had lower ALFF values in some cognitive-related brain regions, such as the left fusiform gyrus, and decreased ALFF in the superior temporal gyrus in the left temporal pole, indicating decreased spontaneous activity in these brain regions, which are closely related to cognitive and memory functions. The fusiform gyrus, located in the occipital-temporal lobe, is involved in advanced visual processing, and is responsible for the recognition of objects, words, and faces. Studies have shown that abnormal functional activity of the fusiform gyrus may contribute to visual cognitive impairment in patients with aMCI (21). The anterior end of the temporal lobe is also thought to be the spiritual cortex, which is associated with the frontal lobe of the brain to co-manage human emotions and mental activities; therefore, injury to the temporal lobe often manifests as cognitive dysfunction (22). The superior temporal gyrus is also a node of the DMN, which is the key to extracting meaningful language features from speech input and is the main coding center in the time domain (23). In addition to speech features, they are regulated by knowledge and perception (24). This study found that decreased ALFF in the temporal pole, the superior temporal gyrus, may be associated with dysfunction of multiple cognitive domains in patients with aMCI. Subsequent correlation analysis confirmed this finding, and the ALFF value of the superior temporal gyrus positively correlated with the MoCA score. The lower the MoCA score and Z value, the worse is the cognitive performance.

An increase in the ALFF indicates an increase in the spontaneous activity of neurons. In this study, the areas of increased ALFF in aMCI included the left cerebellum 8, left inferior temporal gyrus, left superior frontal gyrus (BA11) and right inferior temporal gyrus (BA20). In addition to maintaining balance, muscle tone, and movement coordination, the cerebellum is involved in language, learning, emotion, attention and other cognitive functions. Therefore, studies have shown that typical cerebellar cognitive-affective syndrome is associated with lesions in the aforementioned brain areas (25). The prefrontal cortex, which performs some aspects of cognitive function, information memory and recall, emotional thinking, and perception, is intimately associated with the limbic part of the forebrain and is a key region involved in memory processing in humans (26). Increased ALFF values in the left superior orbital gyrus (BA11) in patients with aMCI are consistent with the hypothesis that AD and MCI patients may use additional neural resources in the prefrontal area to compensate for cognitive function loss (27). Subsequent correlation analysis confirmed these findings, with the left superior frontal gyrus ALFF negatively correlated with the MoCA score. The lower the MoCA score, the higher the Z value, which may be caused by extra neural resources in the prefrontal cortex that compensate for the loss of cognitive function.

### 4.2. Analysis of ReHo index changes in patients with aMCI

ReHo is an imaging measurement that describes the activity of local brain function, and was originally proposed by Zang et al. (28). It reflects the synchronization of voxel time series within each functional area of the brain, localizing areas of difference and observing complex brain functional activities (29). One study used ReHo markers to explore the characteristics of local brain activity in patients with different MCI states (30). The results showed that ReHo values in the left insula increased and decreased in the left superior temporal gyrus during the recovery stage in the MCI group (30). In the stable stage, the ReHo value of the left sub frontal orbital gyrus increased and that of the left inferior parietal lobe decreased in the MCI group (30). In the progressive stage, the ReHo value of the left sub frontal orbital gyrus increased and that of the left putamen decreased in the MCI group (30). This indicates that changes in ReHo values in these specific brain regions provide evidence for active intervention in patients with MCI at different stages.

The increase or decrease in ReHo suggests that the synchronism and coordination mechanism of spontaneous neuronal activity in this brain region may be abnormal, which is helpful for us to better understand brain functional changes related to the disease. In this study, ReHo decreased in the right middle temporal gyrus and right anterior cuneiform lobe. The areas of increased ReHo were the left temporal pole (middle temporal gyrus, left inferior temporal gyrus, cerebellar vermis, right parahippocampal gyrus, left caudate nucleus, right thalamus, and left superior frontal gyrus (BA6)). The frontal lobe is an important part of the brain responsible for higher cognitive function, especially in functional activities such as memory, thinking, and execution. The superior frontal gyrus is located in the upper prefrontal lobe, a region with a relatively complex cellular structure that often collaborates with other brain regions for various cognitive and motor control tasks (31). The medial part of the superior frontal gyrus, which consists of the DMN, is activated in cognitive-related tasks and is critical for learning and memory, acquisition of stress awareness, and integration of thinking and emotional functions (15). Dysexecutive syndromes that primarily affect the frontal lobe result in deficits in the higher-order cognitive processes necessary for effective social functioning, emotional regulation, and motivation (32). Any cause of frontal lobe injury can lead to memory, language, or other forms of cognitive dysfunction. In particular, frontal lobe injury can result in attention and memory disorders, leading to a decline in executive function (33). Godefroy et al. found that brain injury in the frontal cortex is directly associated with executive dysfunction (34). The parahippocampal gyrus is involved in episodic memory, mainly visual associative recognition memory, recall, source memory, and visuospatial processing (35). Research has shown that patients with aMCI have reduced activation in the medial temporal lobe, the extent of which is associated with episodic memory, and that the anterior temporal and frontal lobes work together to manage human emotions and mental activity; therefore, injury to the temporal lobe usually manifests as cognitive impairment (22). In addition, the ReHo values of the cerebellar vermis and the right thalamus increased. The thalamus is an important node in the frontal-subcortical circuitry (36), and its impairment (especially in the thalamus of the dominant hemisphere) can lead to cognitive dysfunction.

Abnormal activity in the cerebellum may affect executive function and speech ability (37). Correlation analysis showed that the ReHo value of the right parahippocampal gyrus was negatively correlated with the MMSE score, and the ReHo value of the right middle temporal gyrus was positively correlated with the MMSE score, which could be used as an imaging index to evaluate cognitive recovery in aMCI. The changes in ReHo values in these brain regions indicate that there are multiple functional impairments in patients with aMCI, which may be related to the dysfunction of multiple cognitive domains.

## 5. Conclusion

The findings of this study suggest that the combination of ALFF and ReHo modalities can comprehensively reveal abnormal brain function in patients with aMCI. Changes in ALFF and ReHo in several cognitive-related brain regions in patients with aMCI suggest that impairment of brain function in specific regions of patients with aMCI is the central mechanism leading to cognitive impairment. It also identified relatively specific brain regions in patients with aMCI and provided targets for early and accurate treatment. This study provides evidence for early clinical diagnosis, dynamic observation of disease course and evaluation of prognosis.

## Data availability statement

The raw data supporting the conclusions of this article will be made available by the authors, without undue reservation.

## Ethics statement

The studies involving humans were approved by Medical Ethical Committee of the First Affiliated Hospital of Heilongjiang University of Chinese Medicine. The studies were conducted in accordance with the local legislation and institutional requirements. The participants provided their written informed consent to participate in this study.

## Author contributions

JY, QZ, L-nC, and X-lL: concept and design. JY, L-nC, D-nC, YW, and X-lL: data curation. L-nC and X-lL: formal analysis. X-lL and SW: funding acquisition. QZ, AL, X-lL, and GY: investigation. JY, W-wZ, and AL: methodology. QZ, AL, X-lL, and GY: project administration. S-wH, XL, YW, D-nC, and X-lL: resources. L-nC: software. QZ, AL, X-lL, and GY: supervision. JY, S-wH, XL, SW, W-wZ, L-nC, D-nC, JM, YH, XC, YW, LC, AL, X-lL, GY, and QZ: validation and visualization. All authors contributed to the article and approved the submitted version.

## Funding

This study was partly supported by the National Foundation of Natural Science of China (82074537 and 81373714); Joint Guidance Project of Natural Science Foundation of Heilongjiang Province (LH2020H103 and LH2021H101); Chinese Medicine Administration of Heilongjiang (ZHY2022-194); and Shanghai Natural Science Foundation Project (19ZR1457800).

## Conflict of interest

JY and QZ were employed by Shenzhen Frontiers in Chinese Medicine Research Co., Ltd. W-wZ was employed by MSD R&D (China) Co., Ltd. AL was employed by Sanofi-Aventis China Investment Co., Ltd.

The remaining authors declare that the research was conducted in the absence of any commercial or financial relationships that could be construed as a potential conflict of interest.

## Publisher’s note

All claims expressed in this article are solely those of the authors and do not necessarily represent those of their affiliated organizations, or those of the publisher, the editors and the reviewers. Any product that may be evaluated in this article, or claim that may be made by its manufacturer, is not guaranteed or endorsed by the publisher.

## Abbreviations

aMCI: amnestic mild cognitive impairment
AD: Alzheimer’s disease
ReHo: Regional Homogeneity
ALFF: amplitude of low-frequency fluctuation
HC: healthy control
MoCA: Montreal Cognitive Assessment
MMSE: Mini-Mental State Examination
